# Methylation as a key regulator of Tau aggregation and neuronal health in Alzheimer’s disease

**DOI:** 10.1186/s12964-021-00732-z

**Published:** 2021-05-07

**Authors:** Abhishek Ankur Balmik, Subashchandrabose Chinnathambi

**Affiliations:** 1 Neurobiology Group, Division of Biochemical Sciences, CSIR-National Chemical Laboratory (CSIR-NCL), Dr. Homi Bhabha Road, 411008, Pune, India; 2 Academy of Scientific and Innovative Research (AcSIR), Ghaziabad, 201002, India

**Keywords:** Tau, Methylation, Methyltransferases, Post-translational modifications, Epigenetics, Aggregation

## Abstract

Neurodegenerative diseases like Alzheimer’s, Parkinson’s and Huntington’s disease involves abnormal aggregation and accumulation of toxic proteins aggregates. Post-translational modifications (PTMs) of the causative proteins play an important role in the etiology of disease as they could either slow down or accelerate the disease progression. Alzheimer disease is associated with the aggregation and accumulation of two major protein aggregates—intracellular neurofibrillary tangles made up of microtubule-associated protein Tau and extracellular Amyloid-β plaques. Post-translational modifications are important for the regulation of Tau`s function but an imbalance in PTMs may lead to abnormal Tau function and aggregation. Tau methylation is one of the important PTM of Tau in its physiological state. However, the methylation signature on Tau lysine changes once it acquires pathological aggregated form. Tau methylation can compete with other PTMs such as acetylation and ubiquitination. The state of PTM at these sites determines the fate of Tau protein in terms of its function and stability. The global methylation in neurons, microglia and astrocytes are involved in multiple cellular functions involving their role in epigenetic regulation of gene expression via DNA methylation. Here, we have discussed the effect of methylation on Tau function in a site-specific manner and their cross-talk with other lysine modifications. We have also elaborated the role of methylation in epigenetic aspects and neurodegenerative conditions associated with the imbalance in methylation metabolism affecting global methylation state of cells.

**Video abstract**

**Supplementary Information:**

The online version contains supplementary material available at 10.1186/s12964-021-00732-z.

## Background

Alzheimer’s disease is associated with the misfolding of majorly two proteins. Amyloid-β peptide aggregates extracellularly, and is generated by the cleavage of membrane-associated amyloid precursor protein (APP). Tau is a key protein involved in stabilization of microtubules in neuronal axons which forms intracellular neurofibrillary tangles (NFTs) [1]. Microtubules functions as the tracks for the molecular motors kinesin and dynein to carry out intracellular transport as well as to clear out accumulation of toxic proteins. Tau malfunction causes a defect in this transport mechanism leading to cytotoxicity and neurodegeneration as they can propagate and induce toxicity in other cells [2–4]. Neurofibrillary tangles are the characteristic hallmark of Alzheimer`s disease and related neurodegenerative Tauopathies in which Tau is the main component [5, 6]. Tau is a highly soluble protein but its abnormal post-translational modifications affect its natively unfolded structure and its ability to associate with microtubules [7–9]. The function and structure of Tau depends on the cellular environment as well as the post translational modifications [10]. Phosphorylation is considered as an important PTM of Tau as it is implicated in both physiological and pathological states. Phosphorylation is required for Tau’s association with microtubules. However, hyperphosphorylation of Tau results in its dissociation from microtubules and leads to aggregation [10–12]. The phosphorylated state of Tau, in turn depends on the level of kinase activity and the balance between kinases and phosphatases in neurons [13]. Mapping of PTMs in Tau protein obtained from AD patient’s brain has revealed phosphorylation sites, which are not present in normal conditions [14]. Some of the major pathological sites include AT8 (pS202/pT205), AT100 (pT212/pS214), AT180 (pT231/pS235), PHF1 (pS396/pS404), pS356, pY394, pT403, pS409 and pS422 [9, 15]. Most of these sites lie within the repeat region and the flanking region (N and C-terminal) of Tau. Modifications at certain sites are likely to induce Tau aggregation by disturbing the charge distribution and altering intramolecular interactions [15–18]. Different families of kinases carry out phosphorylation of Tau. These include proline-directed protein kinases-like GSK-3β, CDK5 and MAP kinases (mitogen-activated protein kinases); non-proline directed protein kinases-like CK (casein kinase), MARKs (microtubule-affinity regulating kinases), PKA (protein kinase A) and tyrosine specific kinases-like SFKs (Src family kinases) [19]. The level and activity of these kinases are elevated in case of AD and most of these are found to be co-localized with NFTs. Tau hyperphosphorylation occurs when there is a net increase in the phosphorylation i.e. there is an imbalance between phosphorylation and dephosphorylation. This condition arises generally due to increase in kinase activity along with inhibition of protein phosphatases. PP2A (Protein phosphatase 2A) is the major phosphatase of the cell with nearly 70% of overall cellular phosphatase activity [20–22]. PP2A is regulated by two modes—methylation and action of endogenous cellular inhibitors called I_1_ and I_2_. PP2A activity may get reduced up to 50% in AD due to hypomethylation or increase in the levels of its inhibitors [23].

It is notable that there are 11 known methylation sites on Tau in physiological conditions while upon aggregation; the extent of methylation is reduced. There are 7 methylation sites that have been mapped in Tau present as paired helical filaments (PHFs) [24, 25]. The methylation at these sites potentially correlates with the occurrence of phosphorylation on serine at these motifs. There have been studies, which showed the association of Tau phosphorylation (pT181) with increased levels of total homocysteine and decreased S-adenosyl methionine: S-adenosyl homocysteine ratio in cerebrospinal fluid (CSF) [26, 27]. Increased homocysteine level is indicative of defective methylation potential in cells. Protein phosphatase 2A (PP2A) functions as an active enzyme in its methylated state which shows the effect of aberrant methylation potential on phosphorylation of Tau [28–30]. Apart from the indirect effect of methylation on Tau phosphorylation, methylation may play an important role in modulation of Tau aggregation propensity. In-vitro Tau methylation has been found to decrease the aggregation propensity of Tau without affecting its ability to stabilize microtubule assembly. Microtubule polymerization was hampered only in the presence of Tau methylated at higher stoichiometries. Methylated Tau formed fibrils similar to unmodified Tau but the overall aggregation propensity and critical concentration of Tau to initiate the aggregation reaction was found to be elevated [24].

**Fig. 1:**
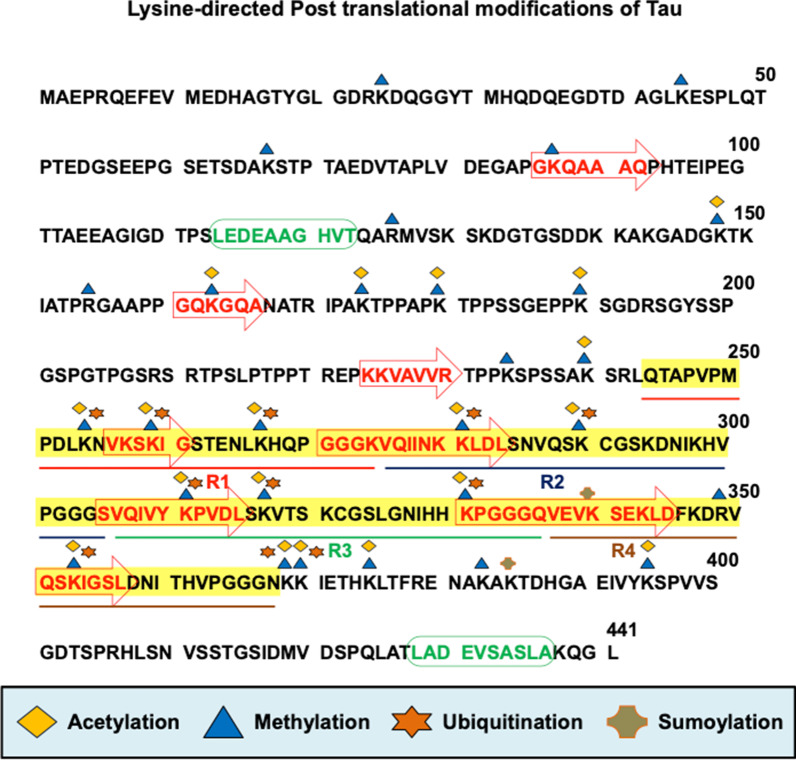
Lysine-directed Post translational modifications of Tau. Tau protein can be subjected to many post-translational modifications owing to its unfolded structure. Tau undergoes post-translational modifications like phosphorylation, acetylation, methylation, etc. which determines its functional state in neurons. Lysine-directed post-translational modifications like acetylation, methylation, ubiquitination and sumoylation also reflects the functional state of Tau as these modifications may compete for a specific lysine residue in Tau with each modification having different effect. For example, ubiquitination marks the protein to direct toward its degradation while other modifications at the same lysine residue may increase its stability

Methylation is carried out by the class of enzymes called methyltransferases. Class II methyltransferases are SET domain-containing enzymes which mainly function as histone methyltransferases [31–33]. However, there are methyltransferases of the same class like G9a and SUV39, which shuttles between nucleus and cytoplasm to act on cytoplasmic proteins [34, 35]. Lysine residues in a protein can be subjected to methylation, acetylation, ubiquitination, SUMOylation and glycation (Fig. 1) [9, 36, 37]. One of the important attributes of post-translational modification at lysine residue is the possibility of competition for modification of a single specific site. The state of modification can determine the function of protein. There is a direct relationship of methylation with other lysine modification mainly acetylation and ubiquitination in Tau protein [9, 25, 29]. The occupancy of a single lysine residue with methylation, acetylation or ubiquitination may drive the fate of Tau protein in different directions. Thus, it is important to study the nature of cross-talks occurring among all these PTMs in order to better understand the mechanism of Tau function in health and disease. Also, there is possible cross-talk between methylation with phosphorylation at PHF6 and PHF6* motifs (VQIINK and VQIVYK), where acetylation seems to play an important role as suggested by some of the studies [38, 39]. However, further exploration is required to understand the underlying mechanisms involved in the cross-talk between methylation and phosphorylation.

## Tau methylation in Alzheimer’s disease

Tau can be subjected to mono-methylation or di-methylation, which determines their regulatory roles, but so far, tri-methylation has not been reported in Tau [9, 24]. For example, the extent of methylation at specific sites is inversely proportional to the aggregation propensity of Tau. Tau methylation occurs at several lysine and a few arginine residues by the action of enzymes called lysine methyl transferases or arginine methyl transferases. However, not much is known about the methyl transferases involved in the modification of Tau protein. There has been a recent report by Bichmann et al., on the role of methyl transferase SETD7 on the Tau mono-methylation at K130 and its nearby lysine residue K132 and its importance in nuclear Tau localization [40]. Most of the methylation sites lie in the microtubule binding region of Tau [9, 24, 25]. In order to access the role of methylation of Tau at MTBR, Funk et al., carried out in vitro tubulin polymerization assay in the absence of Tau and in the presence of synthetically methylated or unmodified Tau. It was observed that Tau methylation does not affect the extent of tubulin polymerization in its methylated state. Tubulin polymerization was found to be depressed only with Tau having higher stoichiometries of methylation. Further, the aggregation propensity of Tau was found to be in inverse relation to the extent of methylation [24].

It has been studied that the extent of mono-methylated sites increases with aging as well as progression of AD. The pool of soluble Tau also contains methylated arginine sites in normal brain. Current knowledge of implications of Tau methylation in AD suggests that methylation is a part of both normal Tau as well as its pathological form as PHFs. Arginine residues R126, R155, and R349 are known to be mono-methylated in both normal and pathological Tau [41]. Arginine methylation in Tau is speculated to be involved in membrane binding of Tau and its nucleo-cytoplasmic shuttling [42, 43]. However, the mechanism of these processes is not clear. Changes in methylation signature occur in AD, which can possibly alter the intramolecular forces within Tau molecule resulting in altered local conformations. The changes in the local conformations in turn affect the solubility and binding properties. Thus, the set of PTMs determines the solubility and aggregation propensity of Tau. Several phosphorylation and methylation sites in Tau are present in close proximity, which may alter the occurrence of both modifications. For example, Tau phosphorylation at S262 was found to occur more frequently along with methylation at K267 [25]. Additionally, methylated Tau was prevalent in the affected regions of brain derived from AD patients. The Tau lesions in AD brain have shown immunoreactivity for methylated Tau when labelled with anti-meK (anti-methylated lysine) antibody [25].

The pattern of methylation on normal Tau and PHF-derived Tau provides an important hint for its regulatory role in aggregation. Normal Tau in human brain can both be mono-methylated or di-methylated while the Tau in PHFs is only mono-methylated [37]. There are eight lysine residues, which are dimethylated out of total eleven methylation sites in Tau. Also, there are fewer methylation sites in PHF-derived Tau as compared to normal Tau. The presence of methylated Tau in the vicinity of phosphorylation sites, especially in the KXGS motifs may provide protective role against phosphorylation. Further, two of the methylation sites at K24 and K44 lie adjacent to caspase and calpain cleavage sites while other generate fragments, which are aggregates prone [44–46]. There are limited studies regarding the direct role of methylation on Tau function and aggregation but the current knowledge suggests that it may have an important role in deciding the fate of Tau.

## Methylation as a mode of epigenetic regulation and its role in Alzheimer’s disease

**Fig. 2:**
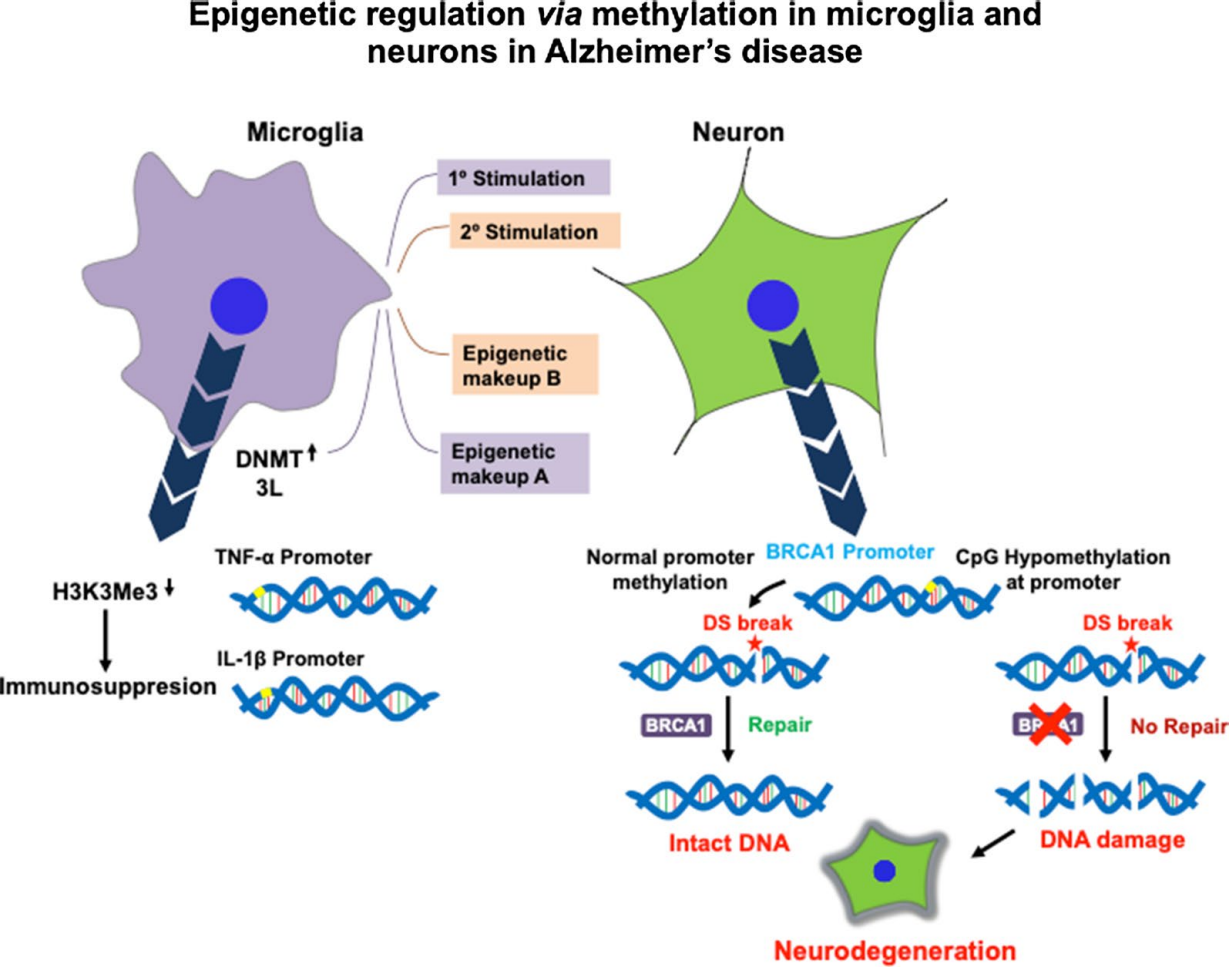
Epigenetic regulation via methylation in microglia and neurons in Alzheimer’s disease. Epigenetic changes in methylation signatures occurs both in microglia and neurons during AD. Gene specific DNA hypomethylation or changes in histone methylation signatures in microglia and neurons result in altered immune function and genomic integrity respectively. Microglia is subjected to stimulation by aggregate species such as oligomers which produce different epigenetic makeups in primed and naïve microglia. DNA methyl transferase DNMT3L has been found to be upregulated in AD after LPS stimulation, suggesting its possible role in microglial activation. On the other hand, hypomethylation at H3K3Me3 on TNF-α and IL-1β promoters is associated with immunosuppression. In case of neurons, CpG hypomethylation occurs at BRCA1 (breast cancer 1) promoter in AD. This results in reduced BRCA1 levels leading to impaired DNA repair leading to neurodegeneration

In neurodegenerative conditions, methylation is involved not only as a PTM of Tau but is also crucial with respect to its role in epigenetic regulation and metabolic aspect. Alzheimer disease is associated with numerous changes in the epigenetic makeup of neural cells including neurons, microglia and astrocytes [47–50]. In microglia, enhancer of zeste homolog 2 (EZH2) works along with the catalytic subunit of polycomb repressive complex 2 in order to carry out transcriptional silencing. This complex is involved in tri-methylation at H3K27 (H3K27me3) [51]. Microglia undergoes frequent changes in its epigenetic makeup and showsphenotypic changes upon stimulation [52]. It has been found that microglia pre-exposed with LPS or TLR4 ligand undergoes distinct changes in epigenetic make-up in its primed and unprimed states [51]. Conversely, under immunosuppressed state, the methylation levels at H3K3Me3 are found to be downregulated. In neuronal cells, CpG hypomethylation at the promoter of brca1 (breast cancer 1) occurs [53]. BRCA1 downregulation results in defects in the double stranded DNA break repair and ultimately leads to neuronal death (Fig. 2). The epigenetic regulation of gene expression occurs through methylation in two ways – modification of lysine residues in histone core and methylation of CpG dinucleotides [54–57]. However, there are occurrences of non-CpG methylation. Both, methylation on histone lysine and DNA methylation serve the purpose of gene silencing and transcriptional suppression. Clusters of CpG called as CpG islands are often present in the promoter and enhancer region of genes. These CpG islands have either methylated or hydroxymethylated cytosines as 5-methyl cytosine (5mC) and 5-methyl hydroxy cytosine (5hmC) [58–60]. 5mC is associated with gene repression while conversion of 5mC into 5hmC represents gene activation [61, 62]. Methylation at CpG hinders binding of transcriptional factors such as Ets-1 as well as host 5mC binding proteins such as MeCP2, MBD1, MBD2 and MBD4, which functions as transcriptional repressor [63]. Apart from DNA methylation at CpG sites, there are also large numbers of CpH (H refers to A, T or C) sites that are methylated [64, 65].

There are five types of DNA methyl transferases involved in the transfer of methyl group from S-adenosyl-L-methionine to nucleotides in DNA – DNMT1, DNMT2, DNMT3a, DNMT3b and DNMT3L [66, 67]. Out of these DNMT1 is primarily involved in the maintenance of methylation signatures on DNA. In Alzheimer’s disease, there are evidences of decreased 5mC levels and DNA methyl transferase 1 (DNMT1) in the hippocampal and temporal brain region [68, 69]. However, in another study, increased levels of DNA methylation and DNMTs have been found in frontal cortex, temporal cortex and cerebellum [70–72]. DNA methylation is a robust mechanism of gene regulation at epigenetic level, such that the methylation signatures changes on gene locus depending on cellular conditions. Methylation levels of CpG islands in enhancer and promoter regions have been studied in AD, which suggest epigenetic dysregulation in enhancers of genes crucial for neuronal health [73, 74]. Loss of methylation at CpH at enhancers and promoters has been observed in AD condition resulting in enhanced target gene expression. The diminished levels of methylation on these target genes are associated with overstimulation of apoptotic and inflammatory pathways [73–76]. Similarly, reduced methylation at bace1 enhancer leads to overproduction of BACE1 which in turn results in amyloid-β production [73, 77]. Upregulation of BACE1 levels are also associated with hypomethylation of enhancer elements in Down syndrome cell adhesion molecule like 1 (DSCAML1). This leads to an excessive upregulation of bace1 in the early stages of AD [73]. Many of the alterations in enhancer methylation lies in the genes regulating the expression of cell cycle regulatory proteins like cyclin-dependent kinases (CDKs). Reduced enhancer methylation of CDKs upregulates their levels and disrupt cell cycle regulation [78–80]. This results in abrupt neuronal cell cycle re-entry which becomes abortive due to lack of proper regulatory mechanisms [79]. This results in the promotion of neuronal death and synaptic loss leading to neurodegeneration. The occurrence of DNA hypomethylation at enhancers is linked with the formation of amyloid-β aggregates in early stages of AD [73].

There are contradictory observations regarding the levels of methylation making it hard to understand the role of DNA methylation in neurodegeneration. Thus, the effect of methylation may be dependent not only on levels but on the locus of DNA methylation. Distinctive patterns of DNA methylation and gene expressions are associated with the normal physiological conditions and in pathological conditions [81–85]. Elaborate study of AD specific methylation signatures on DNA can provide an important biomarker for assessing risk factor, progression and detection of AD.

## Cross talk of Tau methylation with other PTMs

PTMs are the mode of regulation of multiple cellular processes, which themselves are highly regulated. The set of PTMs on a protein generates a code which determines its structure and function. The occurrence of a set of multiple modifications or the probability of a single site being modified by different PTMs is crucial for protein function and varies according to the cellular environment. Various lysine residues on Tau are subjected to more than one kind of modification. For example, K180 can be acetylated or methylated, K254 and K290 can be methylated or ubiquitinated, K385 can be methylated or SUMOylated [9, 36]. The state of PTM on a particular residue is characteristic of Tau functional state.

**Fig. 3:**
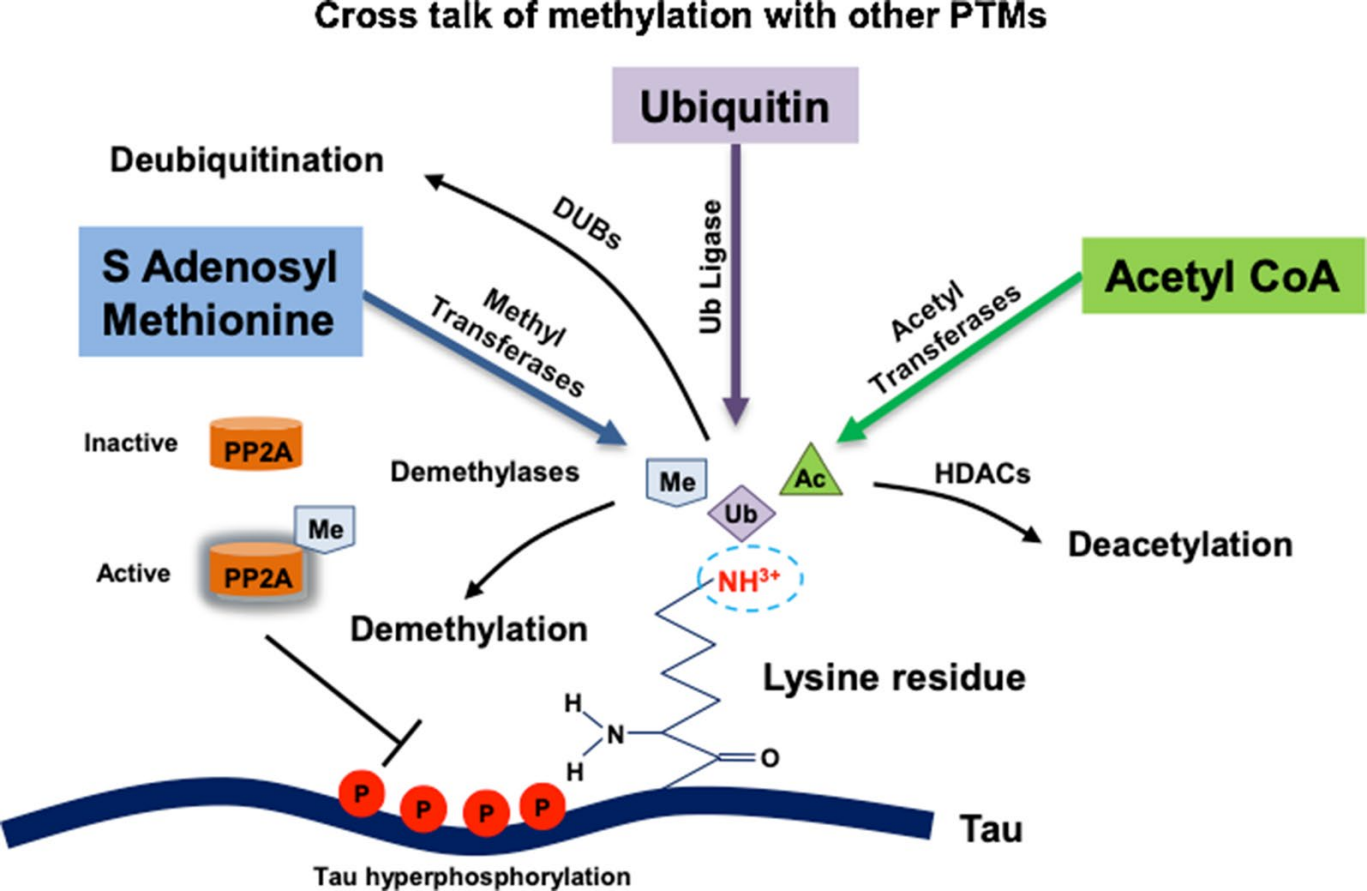
Cross talk of methylation with other PTMs. Cross-talk between methylation, acetylation and ubiquitination may occur over the modification of a specific common lysine residue. Acetyl transferases, methyl transferases and ubiquitin ligase carry out respective modification and works synergistically with histone deacetylases (HDACs), demethylases and deubiquitinating enzymes (DUBs) respectively. Methylation has an indirect cross-talk with phosphorylation as protein phosphatase 2A (PP2A) is activated through methylation and functions to reduce the hyperphosphorylation of Tau

There are evidence for the possible cross-talk between methylation, acetylation, ubiquitination and SUMOylation, with one PTM being preferred as per the condition. Ubiquitination at K254 is critical in physiological condition to maintain Tau homeostasis [25, 86]. In AD, level of Tau methylation at K254 exceeds its ubiquitination level in PHFs, hampering the clearance of Tau aggregates by ubiquitin proteasomal system (UPS) [25]. However, another lysine residue K290 is found to be ubiquitinated in aggregated Tau while methylated in normal conditions [41]. Ubiquitination also has a possible cross-talk with phosphorylation as it is found that Tau ubiquitination in PHFs is associated with phosphorylation as it precedes ubiquitination and incorporation into PHFs [87–90]. Similarly, acetylation as a PTM is known for its role in Tauopathies. Tau protein as PHFs is highly acetylated in pathological state as compared to physiological conditions. Lysine residues K163, K174 and K180 may be subjected to acetylation or methylation in pathological and physiological states respectively [37, 91]. Methylation serves an important function in the stability of Tau protein. There could be a cross-talk between the Tau methylation and phosphorylation, where both sites are adjacent. For example, three of the lysines in KXGS motifs (K259, K290, and K353) are methylated under physiological conditions [24, 37]. Lysine modifications at KXGS motifs greatly reduce the phosphorylation potential on adjacent serine implicating the protective role of methylation. However, lysine acetylation at KXGS motif are found to be present in PHFs and known to increase the hyperphosphorylation of Tau [92]. Most of the sites for methylation are present on microtubule binding region (MTBR), of which three sites overlap with ubiquitination [24, 25]. Acetylation at K163, K174, and/or K180 are reported to occur in vivo, whereas acetylation occupancy increases with progression of AD. Sites within (K274 and K280) or adjacent (K259 and K353) to PHF6* in MTBR are also found to be acetylated [9, 37]. SUMOylation of Tau occurs mainly in two sites – K340 and K385, both of which lie in the repeat domain region of Tau [93]. SUMOylation at K340 is known to have pathological impact as it correlates with Tau phosphorylation at AD-associated phospho-epitopes such as T231 and S262 [94]. Although, SUMOylation at K340 is known to have a pathological role; K385 serves as a site for methylation and ubiquitination as well, suggesting its decisive role in neurodegeneration. The possibility of modification of a single site through distinct PTM labels (methylation, acetylation etc.) can drive towards different fates of Tau protein (Fig. 3). The current evidences of various PTM cross-talks suggest that competition for lysine residues can govern the functional state as well as turnover of Tau protein.

## Regulation of Tau methylation and its metabolic implications in neuronal health

**Fig. 4:**
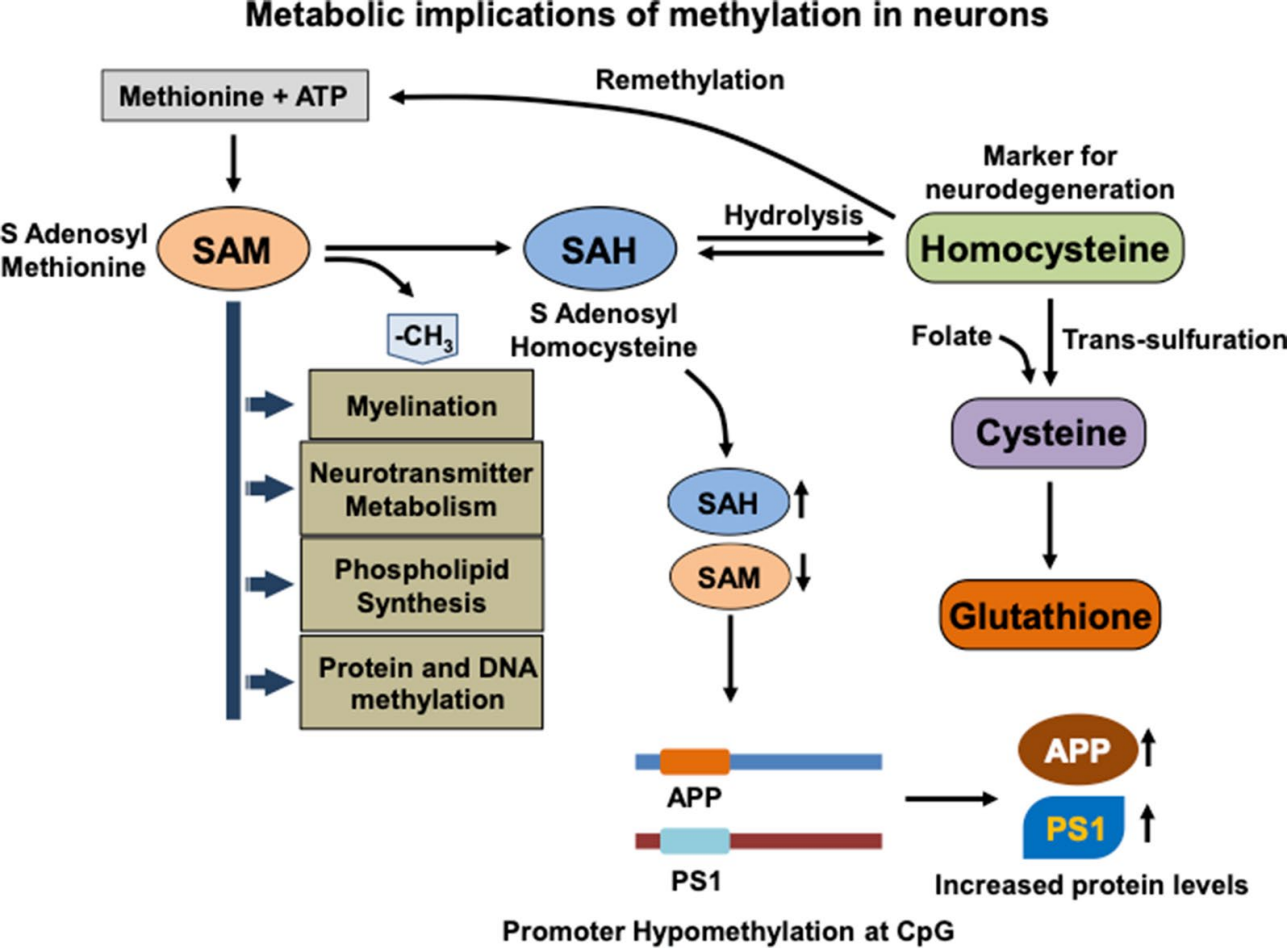
Metabolic implications of methylation in neurons. The state of methylation potential in cells depends on the levels of methionine derived global methylation donor S-adenosyl methionine (SAM) or AdoMet and S-adenosyl homocysteine (SAH). SAM donates methyl group in various cellular processes such as myelination, neurotransmitter metabolism, phospholipid synthesis and protein/DNA methylation. SAH is formed upon methyl donation which gets hydrolyzed to homocysteine in a reversible reaction. Homocysteine can be remethylated in order to replenish the methionine pool or undergoes trans-sulfuration to produce cysteine or gets reconverted into SAH. Thus, the ratio of SAM: SAH determines the overall methylation potential with higher SAM levels favoring methylation. Increased levels of SAH tip the balance towards Homocysteine accumulation in CSF, which can serve as a marker for neurodegenerative conditions. Metabolic conditions which leads to lower SAM:SAH ratio decrease methylation potential in neurons and results in promoter hypomethylation of genes involved in AD viz. amyloid precursor protein (APP) and Presenilin 1 (PS1). Higher APP and PS1 levels gradually lead to protein aggregation and neurodegeneration

The status of overall methylation/demethylation in cell depends on the pool of universal methyl group donor i.e. S-adenosyl methionine (SAM) derived from methionine. SAM, upon donating methyl group gets converted into S-adenosyl homocysteine (SAH), which in turn gets hydrolyzed to homocysteine in a reversible reaction (Fig. 4) [95–97]. Homocysteine can be converted back to methionine by enzyme methionine synthase favoring the optimum methylation potential in cell or get converted into cysteine in trans-sulfuration reaction using folate [98, 99]. Thus, the ratio of SAM and SAH is an important determinant of methylation potential where the higher level of latter reflects disrupted cellular methylation [100]. The metabolism of methyl group in cell is considered a critical factor with respect to neuronal health due to involvement of methylation in various regulatory processes such as gene repression via DNA methylation, epigenetic regulation though histone modification, neurotransmitter metabolism, role in phospholipid synthesis and myelin formation [101–108].

The imbalance in Tau phosphorylation arises either by over activity of kinase or diminished phosphatase activity. In AD, Tau hyperphosphorylation can occur if PP2A activity is suppressed without any alteration of kinase activity [109]. The lower ratio of SAM:SAH is important in Tauopathies, as there is an indirect relationship between disrupted cellular methylation and hyperphosphorylation of Tau [110]. In this aspect, PP2A is an important protein phosphatase known to regulate the phosphorylation state of Tau. PP2A consists of three subunits in its active form—A, B_α_ and C [111–113]. The formation of active enzyme is regulated by reversible methylation on C-terminal of subunit C, which directs the formation of enzyme heterotrimer. Also, methylation occurs on AC dimer, which has been shown to promote its affinity for subunit B_α_ [113]. Thus, methylation plays a central role in PP2A activation. SAH formed as a result of SAM-mediated methylation gives rise to homocysteine, which usually gets converted to methionine or can be reverted back to SAH by associating with adenosine [114]. Certain risk factors like folate (required for trans-sulfuration reaction) or cobalamin (required for conversion of homocysteine into methionine) deficiency, dietary habits, genetic factors etc. promotes SAH accumulation [97, 115–117]. The accumulation of SAH promotes overall hypomethylation favoring the depletion of methyl donor SAM pool as well as by being a competitive inhibitor of methyl transferase enzymes. Increased homocysteine is usually considered a biomarker in vascular diseases [118–120]. Metabolic defects leading to the homocysteine accumulation is known to affect the cognitive function via diverse mechanisms [121, 122]. Homocysteine is responsible for affecting neuronal health via oxidative stress, amyloid-β deposition and promoting Tau phosphorylation [123–130]. Homocysteine levels can be considered both as a risk factor and a pathological marker. Thus, targeting the elevated homocysteine level may aid in checking the progression of AD.

## Summary and future directions

The occurrence and progression of Alzheimer’s disease is dependent on a myriad of factors, of which, post-translational modifications of key proteins play a major role. Tau is subjected to a large numbers of PTMs at multiple sites and in terms of PTMs of Tau, phosphorylation is well-studied and found to have a definitive role in disease progression. However, the role of methylation needs to be explored and understood clearly. On the one hand, Tau methylation serves protective function against its aggregation while on the other, it may have deleterious effect. Depending on the site of methylation and its possible cross-talk and competition for available site, the effect can vary. Lysine residues that can be subjected to both acetylation and methylation are important with respect to Tau function and stability as acetylation is known to be associated with aggregated Tau. Tau PHFs derived from AD brains are heavily acetylated at multiple sites. The protective function of methylation against Tau aggregation can be attributed to preferential methylation of such sites. However, lysine residues like K254 which can be subjected to methylation and ubiquitination, presents a different scenario. In such case, methylation may hinder Tau degradation and turn-over in cells by hampering the proteasomal degradation of Tau.

The epigenetic regulation is an important aspect of Alzheimer’s disease as the expression level of many key proteins such as APP, BACE1, Presenilins and ApoE are known to be under epigenetic regulation. Here, methylation’s role as a gene repressor via DNA methylation as well as in chromatin remodelling through histone lysine modification is crucial. The overall methylation potential of cell is required for controlling the transcriptional levels of genes. The conditions that promote hypomethylation could lead to enhanced levels of gene transcripts and thus, increased protein levels. The proteins that are directly (APP, Tau, and Presenilins) or indirectly (BACE1 and various other kinases) involved in AD progression are upregulated resulting in shifting the equilibrium towards disease progression. Further, the enzymes involved in protective function such as PP2A are regulated via methylation. Under diminished methylation in cell, PP2A suppression leads to increased and abnormal levels of phosphorylation including hyperphosphorylation of Tau.

Methylation is involved directly in Tau regulation as well as epigenetic mechanisms and hypomethylated state in cells is one of the causative factors. There is an intricate balance between the level of universal methyl group donor SAM and its counterpart SAH which determines the total methylation potential. Methyl group metabolism imbalance may be caused by both intrinsic and extrinsic factors, resulting in lower SAM:SAH ratio and thus reduced methylation potential. In such case, the levels of plasma homocysteine is highly elevated which had been used as a marker for cardiac health for a long time. However, its level is also found to be elevated in neurodegenerative conditions suggesting the important role of methylation.

Methylation can serve as a repressor or activator of gene expression depending on the site of histone lysine modification [131]. Administration of specific DNMT inhibitors can help alleviate pathological conditions that arise from hypermethylation. Hypoxic conditions in cortical and hippocampal neurons resulted in increased H3K9Me2 and decreased H3 acetylation on neprilysin promoter leading to its downregulation. Reduced neprilysin levels promote amyloid-β plaque accumulation since it functions as an Aβ degrading enzyme [132]. Diazepinquinazolin-amine derivative-BIX-01294 is a DNMT inhibitor which specifically acts on methyl transferase G9a [133]. BIX-01294 treatment has been reported to replenish synaptic plasticity in amyloid-β rat model [134]. However, most of the inhibitors or modulators of methylation such as decitabine (DAC) and azacitidine (AZA), are non-specific in nature and shows global genome wide effect [135]. Thus, the employing inhibitors or modulators which are specific in nature or agents which can work to maintain the methylation potential are desirable to design therapeutic strategies.

**Fig. 5:**
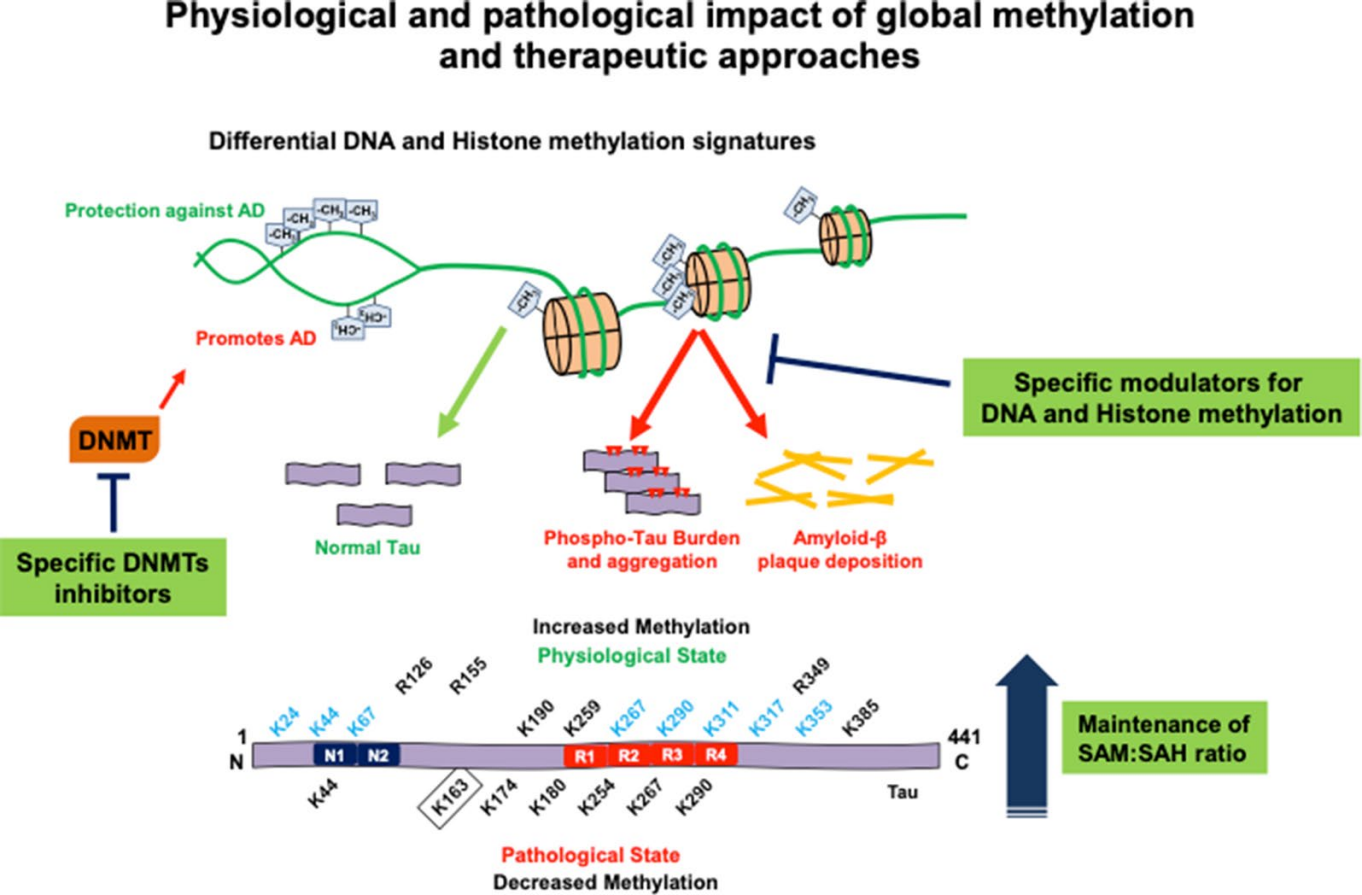
Physiological and pathological impact of global methylation and therapeutic approaches. Tau protein mapped for methylation in physiological and pathological state has shown reduced methylation in pathological conditions. During Alzheimer disease, the overall methylation potential of neurons get reduced, which affects Tau function as well as regulation at epigenetic level. There are 14 methylation sites in normal state, out of which 8 sites can be dimethylated (shown in blue) while only 7 Tau methylation sites are found in Alzheimer disease. On epigenetic level, differential methylation signatures on histones as well as DNA leads to increase in phosphorylated Tau burden, Tau aggregation and amyloid-β deposition. However, methylation is highly regulated modification and functions both in gene silencing and activation. Thus, methylation as an epigenetic regulator can be both protective and deleterious in case of AD. Therapeutic strategies employing the specific DNMT inhibitors and modulators of histone and DNA methylation may prove to be useful against AD. On the other hand, aberrant Tau and/or DNA/histone methylation involves deregulation of one carbon metabolism lowering the SAM: SAH ratio. Strategies to replenish SAM: SAH ratio can aid to protect neurons against oxidative stress, aggregate burden and deleterious effects of Tau and DNA hypomethylation

Dietary habits and therapeutic intervention can help in restoring the normal homocysteine levels and thus methylation potential. Since, methylation is involved both directly as Tau modifier and indirectly as epigenetic modulator on AD; it can prove to be an important therapeutic target for disease prevention. Alzheimer’s disease is associated with the lower levels of SAM as seen in the AD brain [136, 137]. In AD, changes in the one-carbon metabolism involving methylation are evident, hampering the global methylation potential. Diminished methylation potential in turn results in overall hypomethylation. Hypomethylated state in neurons is associated with Tau aggregation, increased Presenilin expression and amyloid-β accumulation [138, 139]. Thus, therapeutic strategies aimed to replenish the diminished methylation potential in neurons may prove to be beneficial in the treatment of AD **(**Fig. 5**)**. Administration of SAM in 3xTg-AD mice was found to be effective against amyloid-β and Tau pathology and ameliorates the factors associated with AD such as genetic predisposition and oxidative stress [140, 141].

Natural compounds able to modulate the state of DNA methylation may provide an accessory approach to alleviate pathological hallmarks in AD. For example, Epigallocatechin-3-gallate (EGCG) competitively inhibits DNMT1 and results in re-expression of gene silenced via DNMT1-mediated methylation [142–144]. There are other small molecules of natural origin such as—naringin, apigenin, luteolin, curcumin, genistein etc., known to have moderate effects on DNA methylation [144–146].
